# Prognostic Value of PCR Cycle Threshold Value in Crimean-Congo Hemorrhagic Fever, Iraq, 2022–2023

**DOI:** 10.3201/eid3207.251284

**Published:** 2026-07

**Authors:** Raghad I. Khaleel, Ihab R. Aakef, Riyadh A. Al-hilfi, Hussein A. Hasan, Iman M. Aufi, Hawraa A. Shakir, Ahmed A. Hussein, Noora A. Abdulhadi, Zainb A. Mohsin, Amal A. Raheem, Sarah W. Ahmed, Ghazwan A. Baghdadi, Chiori Kodama, Antoine Chaillon, Anaïs Legand, Pierre Formenty, Sinan G. Mahdi, Adnan Khamasi

**Affiliations:** Central Public Health Laboratory, Baghdad, Iraq (R.I. Khaleel, H.A. Hasan, I.M. Aufi, H.A. Shakir, A.A. Hussein, N.A. Abdulhadi, Z.A. Mohsin, A.A. Raheem, S.W. Ahmaed); Communicable Disease Control Center, Baghdad (I.R. Aakef, G.A. Baghdadi, S.G. Mahdi); Directorate of Public Health, Ministry of Health, Baghdad (R.A. Al-hilfi); World Health Organization Eastern Mediterranean Regional Office, Cairo, Egypt (C. Kodama); World Health Organization Emergencies Programme, Geneva, Switzerland (A. Chaillon, A. Legand, P. Formenty); World Health Organization Country Office, Baghdad (A. Khamasi)

**Keywords:** Crimean-Congo hemorrhagic fever, viruses, zoonoses, vector-borne infections, PCR, cycle threshold, Iraq, outbreak, laboratory

## Abstract

Using laboratory and epidemiologic data collected in 2022 and 2023 in Iraq, we aimed to evaluate the diagnostic and prognostic performance of reverse transcription PCR (RT-PCR) in Crimean-Congo hemorrhagic fever (CCHF) patients and to identify factors associated with disease outcomes. CCHF was confirmed in 955 hospitalized patients. Among those, RT-PCR analysis showed that blood specimens from deceased patients had a lower median cycle threshold (Ct) value than did those who recovered; we used those data to determine a cutoff value. Univariate and multivariate logistic regression analysis indicated that low Ct values, hemorrhagic symptoms at admission, and age >15 years were independent determinants of fatal CCHF outcome. Viral load and patient age play key roles in the outcome of CCHF in Iraq. Ct value at admission, as a proxy for viral load, serves as a practical indicator to guide clinicians in managing CCHF patients.

Crimean-Congo hemorrhagic fever (CCHF) is a severe tickborne disease caused by CCHF virus (CCHFV; family Nairoviridae, genus *Orthonairovirus*) (*1*). CCHF is endemic across Africa, southern and eastern Europe, the Middle East, and countries in Asia south of the 50th parallel north, which represents the geographic limit of detection of the principal vector and natural reservoir, the *Hyalomma* species tick (*2*,*3*). Reported case-fatality rates (CFRs) vary by region and country, from 5% to as high as 30% (*4*). CCHFV is primarily transmitted to humans through bites of infected ticks or direct contact with blood or tissues of viremic livestock or infected individual animals (*5*,*6*).

As of June 2026, no antiviral drug or licensed vaccine is available for CCHF; however, several therapeutic and vaccines candidates are under development. Outbreak control primarily relies on preventing transmission through isolating persons with suspected and confirmed CCHF, early detection via surveillance and laboratory diagnostics, intensive supportive care, infection prevention and control in health facilities, and community engagement (*7*).

CCHF cases were first reported in 1979 in Iraq; ≈50 cases were detected annually during 1986–1996. Reported cases declined during 1998–2020 (*8*–*10*), but major outbreaks occurred in 2022 and 2023 (*11*–*13*).

The Central Public Health Laboratory (CPHL) viral hemorrhagic fevers unit, CCHF laboratory (Baghdad, Iraq), serves as the national reference laboratory for CCHF diagnosis in Iraq. The Iraq Communicable Disease Control Center (Iraq-CDC) coordinates the national response to CCHF outbreaks. The Iraq-CDC has developed an epidemiologic database to analyze data and guide control and prevention interventions. Here, we present an analysis of CPHL-generated data from 2022–2023, in conjunction with epidemiologic data collected by Iraq-CDC. Patients’ data were recorded as part of Ministry of Health guidelines on CCHF management. We conducted the study under the supervision of the Ministry of Health of Iraq and with appropriate ethics approval obtained from the MOH. We used anonymized data for analysis.

## Methods

### Patients and Specimens

The Iraq-CDC case deﬁnition for a suspected CCHF case was as follows: any person with acute onset of fever with >1 of the following symptoms: headache, backache, joint pain, stomach pain, vomiting; and with history of exposure to one of the following risk factors within the previous 14 days: contact with animal, animal tissues or fluids during slaughtering, raw meat during preparation; or tick bite history or involvement in the removal of a tick from a person or an animal; or contact with a suspected or confirmed case of CCHF. A blood specimen was collected from live patients with suspected CCHF to establish the diagnosis. All suspected and laboratory-conﬁrmed CCHF cases were admitted to general hospitals across governorates in Iraq and managed in their intensive care units.

Blood samples were collected from patients with suspected CCHF at hospital admission; they were admitted into the specialized unit for patients with suspected CCHF. Samples were shipped to CPHL and tested by reverse transcription PCR (RT-PCR). Blood samples from patients with negative RT-PCR results were retested by ELISA IgM serology.

### Diagnostic Assays

Blood specimens were collected on gel and clot activator tube. After separation, 10 mL serum from each specimen was transferred to plain tubes and transported, cooled in ice, to the CPHL in Baghdad for diagnosis. We extracted viral RNA from whole serum samples by using the QIAamp viral RNA mini kit (QIAGEN, https://www.qiagen.com), according to the manufacturer’s instructions.

We performed RT-PCR testing using the RealStar CCHFV RT-PCR Kit 1.0 detection kit (Altona Diagnostics GmbH, https://www.altona-diagnostics.com), which targets the small segment of the CCHFV virus, on the QuantStudio 5 PCR thermocycler (ThermoFisher, https://www.thermofisher.com), according to the manufacturer’s instructions. We added the internal control template of the kit to the sample before RNA extraction; only results with a valid run control were communicated. In brief, the protocol runs as a reverse transcription holding step at 50°C for 10 minutes, denaturation holding phase at 95°C for 2 minutes, then 45 cycles of amplification each at 95°C, 55°C, and 72°C. We used 10 μL of extracted RNA per reaction for a final volume of 25 μL. Assay conditions were consistent throughout the study period, using the same diagnostic kit, standardized protocol, and calibration procedures. We used ELISA IgM serology test (Abbexa LTD, https://www.abbexa.com) in accordance with manufacturer’s protocol to determine levels of CCHFV IgM in the serum of suspected patients with negative RT-PCR results.

### Data Management

General hospitals and Iraq-CDC provided patients’ demographic data, included on the laboratory request form accompanying each sample. Laboratory results were provided to Iraq-CDC and the hospital by CPHL. Data were captured in both CPHL’s and Iraq-CDC’s databases (Microsoft Excel, https://www.microsoft.com). Variables included age, sex, governorate of residence, hospital patient identiﬁer, sample identiﬁer, sample type, collection date, date of symptom onset, patient’s outcome, date of death, laboratory result, and cycle threshold (Ct) values in patients who tested positive by RT-PCR.

To validate the demographic information and document disease outcome, we merged the CPHL and Iraq-CDC databases using hospital patient and sample identiﬁers recorded in both databases. We resolved all inconsistencies through thorough investigation, examining all available records. A full dataset was not available for 13 confirmed cases that are not included in this report.

We classiﬁed patients into 2 main groups for analysis: non-CCHF cases, defined as suspected cases of CCHF with subsequent negative CCHF results by RT-PCR and negative IgM serology; or CCHF confirmed cases, defined as suspected CCHF cases with subsequent positive RT-PCR result, positive IgM serology, or both. We excluded patients with missing or conflicting data that prevented classification into either category from the analysis. The ﬁnal database comprised 3,546 patients; all 3,546 samples were collected during January 2022–December 2023.

### Statistical Analysis

We described categorical variables as percentages. The denominator varied between variables because of missing data. We described continuous variables by medians and interquartile range (IQRs) and associations between independent variables and the dichotomous outcome (survival or death) with crude (unadjusted) odds ratios (ORs).

We used logistic regression models to investigate factors associated with patient outcomes and hemorrhagic conditions. We conducted initial analyses using univariate regression to explore the relationship between individual predictors and the outcome variable. We used univariate analysis to explore unadjusted associations between variables and outcome and used ORs and 95% CIs to quantify the strength of those associations. We considered a p value <0.25 and other variables of known clinical relevance (including those not statistically significant in univariate analysis) for inclusion in the multivariable model. We then used multivariate logistic regression models to adjust for potential confounders. The final model included the following covariates: Ct value, sex, age, hemorrhagic signs, time from onset to admission, tick bite, contact with a confirmed patient, contact with raw meat, contact with animal, and year of outbreaks. We assessed model fit using the Akaike Information Criterion and likelihood ratio tests. We defined statistical significance as p<0.05. We performed all analyses in R version 4.4.1 (The R Project for Statistical Computing, https://www.r-project.org) using the stats (https://www.rdocumentation.org/packages/stats/versions/3.6.2) and gtsummary (https://www.danieldsjoberg.com/gtsummary) packages. We conducted receiver operating characteristic (ROC) analysis to determine the optimal Ct cutoff for predicting patient outcome using the pROC package in R (*14*,*15*).

## Results

### CCHF Confirmation by Testing Methods

**Figure 1:**
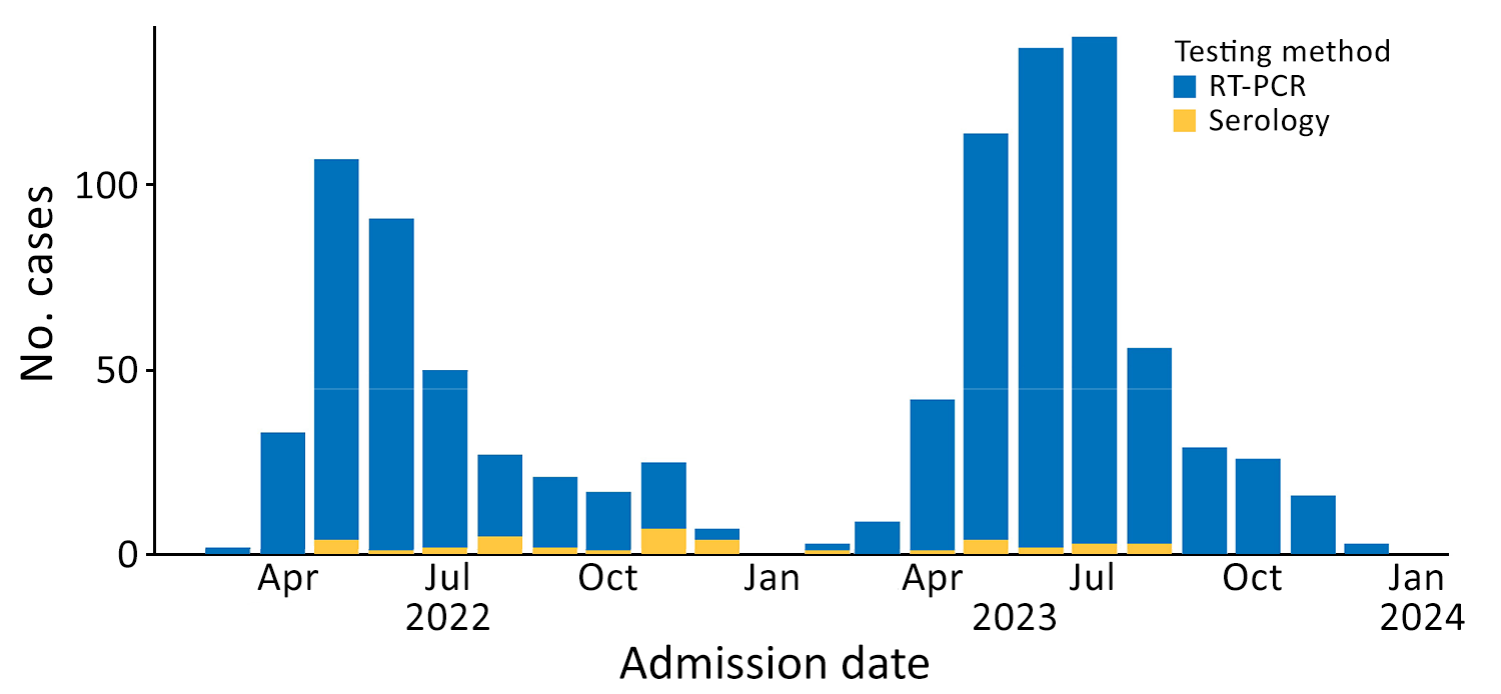
Distribution of confirmed Crimean-Congo hemorrhagic fever patients by testing method in study of prognostic value of PCR cycle threshold value for Crimean-Congo hemorrhagic fever, Iraq, 2022–2023. RT-PCR, reverse transcription PCR.

Our analysis includes a total of 3,546 suspected CCHF patients who sought care at hospitals in Iraq during 2022–2023. CCHF infection was conﬁrmed in 955 (27%) suspected cases; 915 were confirmed by RT-PCR, and an additional 40 that were negative by RT-PCR were confirmed by IgM ELISA (Table 1; Figure 1). In 2022, among 1,370 symptomatic suspected cases, CCHF infection was confirmed in 380 (27%) patients, 354 confirmed by RT-PCR and 26 by IgM ELISA. In 2023, among 2,176 symptomatic suspected cases, CCHF infection was confirmed in 575 (26%) patients, 561 confirmed by RT-PCR and 14 by IgM ELISA. 

**Table 1:** Characteristics of CCHF-confirmed patients in study of prognostic value of PCR cycle threshold for Crimean-Congo hemorrhagic fever, Iraq, 2022–2023

Characteristic	Values		p value†
	2022	2023	
Age			0.10
<15	17/380 (4.5)	16/575 (3)	
15–24	99/380 (26)	133/575 (23)	
25–34	61/380 (16)	131/575 (23)	
35–44	85/380 (22)	130/575 (23)	
45–54	73/380 (19)	92/575 (16)	
>55	45/380 (12)	73/575 (13)	
Median (range)	35 (1–90)	35 (2–80)	
Sex			0.35
F	149/380 (39)	243/575 (42)	
M	231/380 (61)	332/575 (58)	
Residency status			1.00
Rural	1/1 (100)	262/575 (46)	
Semiurban	0/1 (0)	81/575 (14)	
Urban	0/1 (0)	232/575 (40)	
Ct value, median (range)	25.8 (11.4–36.9)	27.4 (12.9–38.9)	<0.001
Exposures and clinical features			<0.001
Median time from onset to admission, d (range)	3 (0–13)	4 (0–20)	
Fever	359/380 (94)	567/575 (99)	<0.001
Contact with confirmed case	18/375 (5)	15/567 (3)	0.08
Contact with animals	212/375 (57)	297/575 (52)	0.14
Slaughtering	127/375 (34)	315/574 (55)	<0.001
Contact with raw meat	207/375 (55)	359/575 (63)	0.02
Tick bite	134/374 (36)	42/575 (7)	<0.001
Hemorrhagic	145/380 (38)	195/575 (34)	0.18
Ecchymosis	104/380 (27)	134/575 (23)	0.16
Bleeding at injection site	109/379 (29)	145/575 (25)	0.23
Injected eyes	1/1 (100)	41/575 (7)	0.07
Ribavirin	1/1 (100)	190/535 (36)	0.36
Outcome			0.02
Cured	305/380 (80)	494/575 (86)	
Death	75/380 (20)	81/575 (14)	

*Values are no. (%) except as indicated, Percentages may not sum to 100% due to rounding. Ct, cycle threshold †Determined by Pearson χ^2^ test, Wilcoxon rank-sum test, or Fisher exact test.

### Admission and Rate of Confirmation by Month

In 2022–2023, among all suspected cases with available month of admission data (n = 3,514), a total of 3,096 (88%) were admitted during April–September (Figure 1). During that period, mean positivity rate was 29% (median 27%, range 22%–41%). Outside of the period, mean positivity rate was 19% (median 23%, range 0%–31%) (Appendix Figure 1). Among the 955 confirmed cases, 848 (89%) patients were admitted during April–September. The average number of confirmed patients admitted per month during April–September was 141, whereas during October–March, the monthly average was 21.

### CFR over Time

**Figure 2:**
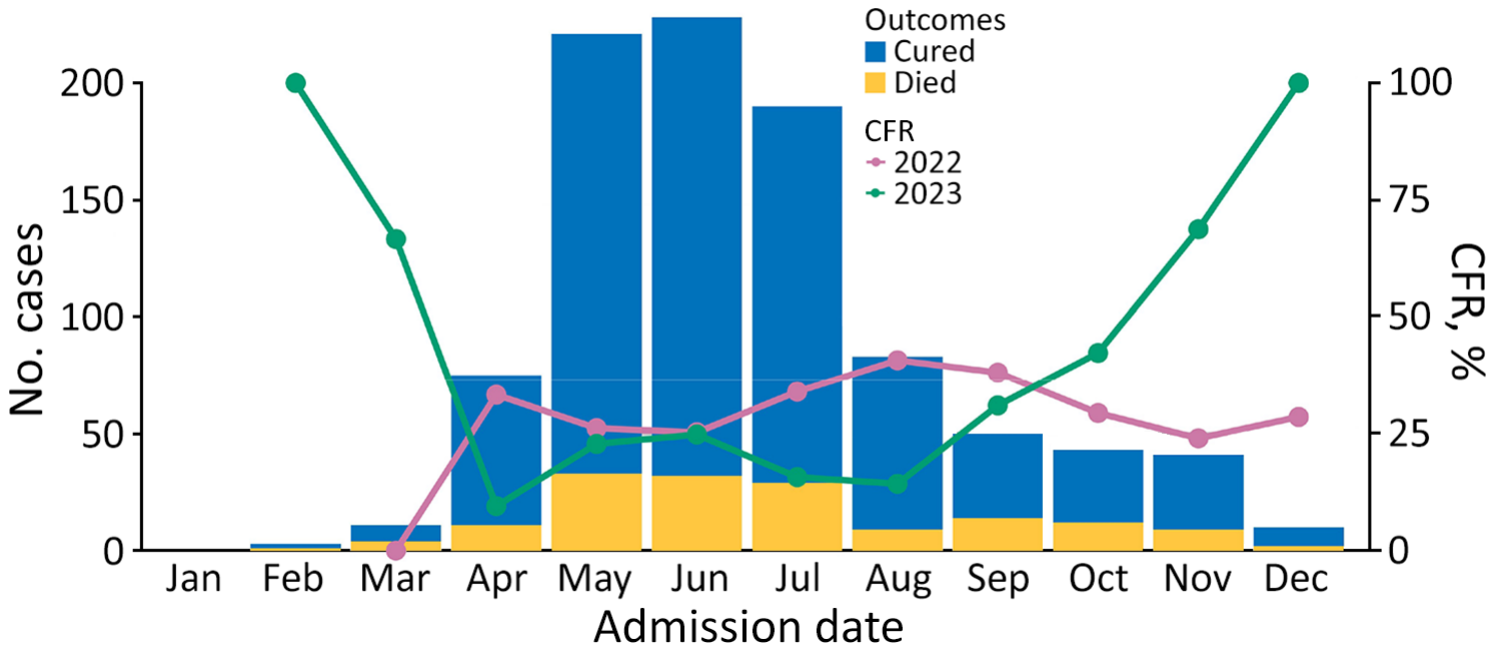
Confirmed Crimean-Congo hemorrhagic fever cases and CFRs by month in study of prognostic value of PCR cycle threshold for Crimean-Congo hemorrhagic fever, Iraq, 2022–2023. CFR was reported if >5 cases were confirmed in that month. CFR, case-fatality ratio.

In 2022–2023, the CFR of confirmed CCHF hospitalized cases was 16%. The mean monthly CFR among conﬁrmed cases was 22% (median 17%, range 10%–50%). During April–September, the mean monthly CFR among confirmed cases was 15% (median 15%, range 10%–21%), whereas during October–March, the mean monthly CFR among confirmed cases was 27% (median 21%, range 14%–50%) (Figure 2).

### Distribution of Cases and CFRs by Governorate

**Figure 3:**
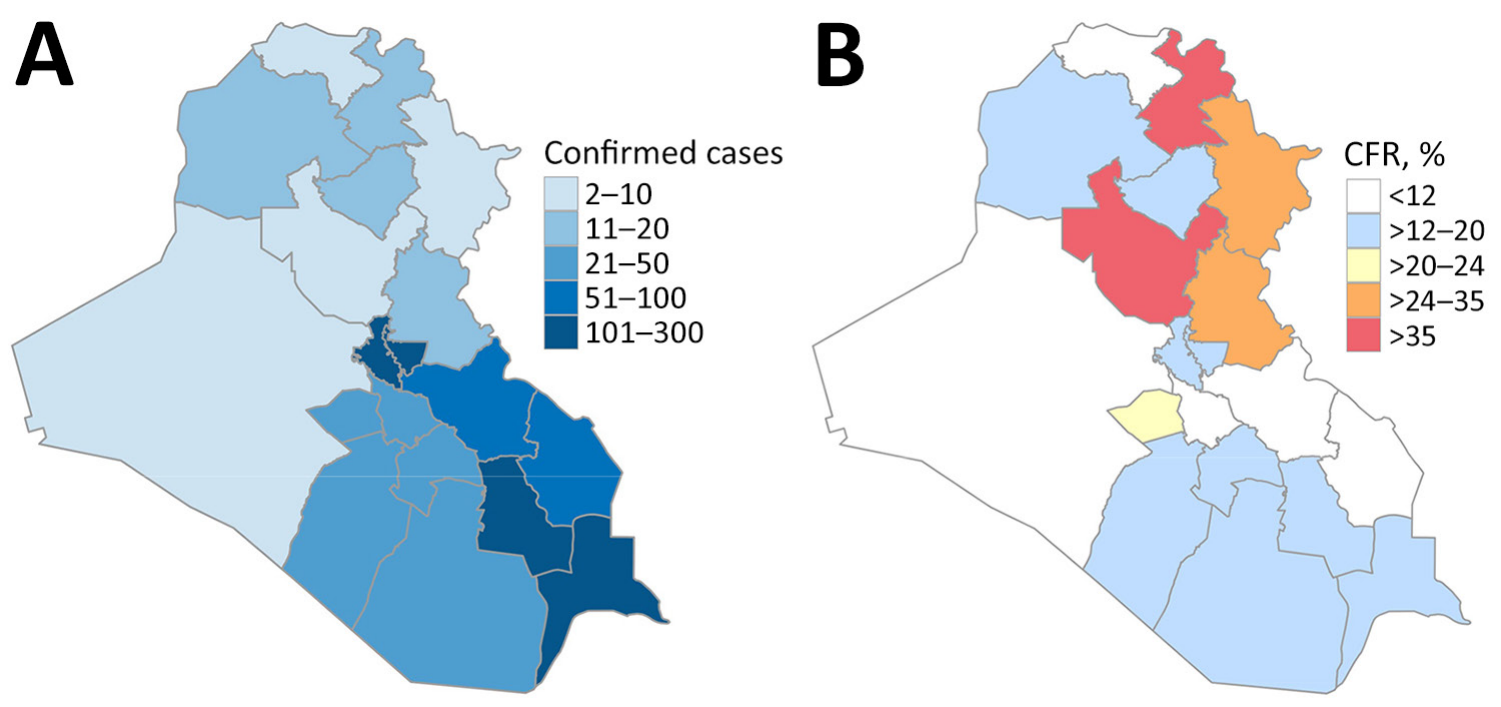
Distribution of confirmed Crimean-Congo hemorrhagic fever (CCHF) patients and CFRs by governorate in study of prognostic value of PCR cycle threshold for CCHF, Iraq, 2022–2023. A) CCHF-confirmed patients by governorate. B) CFR among confirmed CCHF patients by governorate. CFR, case-fatality ratio.

In 2022–2023, confirmed CCHF cases were admitted in hospitals of 19 governorates. Three governorates accounted for most cases, reporting a combined total of 523 (55%) cases; Thi-Qar reported 302 (32%), Baghdad reported 116 (12%), and Basra reported 105 (11%). Babylon, Missan, Muthana, and Wassit governorates reported 5%–8% of all confirmed cases (Figure 3).

CFR varied by governorate; the range was 0%–41% (Figure 3). CFR decreased from 2022 to 2023 in all but 4 governorates (Appendix Figure 2). There was no statistical difference in Ct value at admission across governorates (Appendix Figure 3).

### Ct Value and Outcome

**Figure 4:**
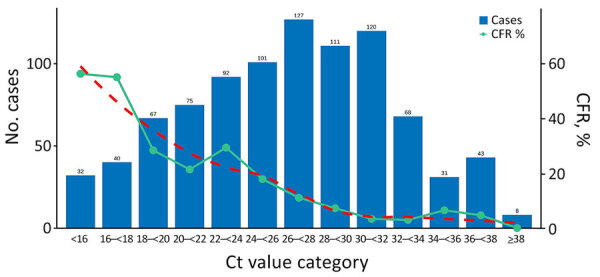
Distribution of confirmed Crimean-Congo hemorrhagic fever patients (n = 915) by Ct value category and corresponding CFR in study of prognostic value of PCR cycle threshold for Crimean-Congo hemorrhagic fever, Iraq, 2022–2023. Case fatality ratio is plotted for each Ct values category, indicating an inverse correlation between Ct value and outcome. Red dashed line shows the smoothed CFR trend across categories (p = 3.28 × 10^−5^; locally estimated scatterplot smoothing fit, p<0.05). CFR, case-fatality ratio; Ct, cycle threshold.

Of the patients with CCHF diagnosis, 915 had their diagnosis confirmed by RT-PCR. RT-PCR analysis of blood specimens from patients who died yielded a lower median Ct value (22.1 [range 12.9–37.0]) than those from patients who recovered (27.7 [range 11.4–38.9]). When plotting the CFR versus Ct categories to assess the relationship between Ct value and outcome, we noted an inverse correlation between Ct and CFR (p<0.001) (Figure 4). That finding suggests that the Ct value at admission has a strong prognostic value. We performed ROC analyses to determine the optimal Ct value to predict participant outcome, using an area under the curve value of 0.76 (Appendix Figure 4); we determined an optimal Ct cutoff of 26 by ROC analysis by Youden method for predicting patients’ outcome (sensitivity 81.6%, specificity 60.9%). The median Ct value on admission to the hospital showed no trend over time (Appendix Figure 5), but median Ct value was overall lower in 2022 than in 2023 (25.8 vs. 27.4; p<0.001) (Figure 1).

### Age and CFR

Among patients <15 years of age (n = 33), median Ct value was 29.9 (range 13.9–38), and no deaths were reported. In older patients (n = 922), CFR fluctuated from 13% to 19%; median Ct value was 26.7 (range 11.4–38.9) (Appendix Figures 6, 7).

### Logistic Regression Analysis

We used logistic regression models to determine factors associated with fatal outcomes. We performed a univariate analysis to explore unadjusted associations between laboratory (Ct value) and relevant demographics (age, sex), clinical and epidemiologic data (hemorrhagic signs, time from onset to admission, tick bites, contact with confirmed cases, animal or raw meat, year of outbreak), and patient outcomes. Characteristics associated with an unfavorable outcome included lower Ct value (OR 0.83 [95% CI 0.8–0.86]; p<0.001), hemorrhagic signs (OR 2.18 [95% CI 1.54–3.09]; p<0.001), and age (OR 1.01 [95% CI 1.0–1.02]; p = 0.041). Receiving a diagnosis in 2023 was also associated with a lower risk for a fatal outcome (OR 0.67 [95% CI 0.47–0.94]; p = 0.022). Those results remained significant for all but age and year of outbreak after false discovery rate correction. Sex; contact with confirmed cases, animal, or raw meat; and history of tick bites were not associated with an unfavorable outcome in the univariate analyses (p>0.5); therefore, we removed those variables from the multivariate analyses (Appendix Table 1). Characteristics associated with a higher risk for unfavorable outcome by multivariate modeling included lower Ct (OR 0.82 [95% CI 0.79–0.86]; p<0.001) and hemorrhagic signs (OR 2.75 [95% CI] 1.86–4.08; p<0.001) (Table 2).

**Table 2:** Multivariate logistic regression results from study of prognostic value of PCR cycle threshold value for Crimean-Congo hemorrhagic fever, Iraq, 2022–2023*

Characteristic	OR (95% CI)	p value	q value†	
Ct	0.83 (0.79–0.86)	<0.001	**<0.001**	
Hemorrhagic		<0.001	**<0.001**	
N	NA			
Y	2.72 (1.82–4.07)			
Age	1.01 (1.00–1.02)	0.094	0.31	
Sex		0.73	0.92	
M	NA			
F	0.93 (0.62–1.39)			
Time from onset to admission	0.97 (0.89–1.06)	0.56	0.79	
Tick bite		0.98	0.98	
N	NA			
Y	1.0 (0.56–1.73)			
Contact with confirmed case		0.38	0.71	
N	NA			
Y	0.61 (0.17–1.77)			
Contact with raw meat		0.38	0.71	
N	NA			
Y	1.20 (0.80–1.80)			
Contact with animal		0.86	0.96	
N	NA			
Y	1.04 (0.68–1.57)			
Year of outbreak		0.42	0.71	
2022	NA			
2023	0.84 (0.55–1.29)			

*We analyzed data from 896 cases confirmed by reverse transcription PCR. A total of 150 of those case-patients were deceased. Bold text indicates statistical significance after false discovery rate correction. Ct, cycle threshold; NA, not applicable; OR, odds ratio. †False discovery rate correction for multiple testing.

## Discussion

### CCHF Trends in 2022 and 2023

Our study observed a clear seasonal pattern consistent with previous reports (*13*); during April–September, 89% of all patients with confirmed cases were admitted to a hospital. Similar trends have been documented in studies on tick seasonality in southern Iraq (*16*), where tick populations increased from spring to autumn, whereas winter months recorded the lowest rates. The observed seasonal variation in tick density appears to correlate with the frequency of CCHF cases. However, further research is needed to better understand the factors contributing to the apparent population increase of the primary CCHFV vector, ticks of the genus *Hyalomma*, and to identify effective control measures.

We observed notable differences between 2022 and 2023. The overall CFR was higher in 2022 than in 2023 (20% vs. 14%), despite a greater number of reported cases in 2023. In addition, median Ct value was lower in 2022 than in 2023 (25.8 vs. 27.4). In 2023, the median monthly CFR was lower during April–September than during the rest of the year (12% vs. 32%). That pattern contrasts with 2022, when the median monthly CFR fluctuated from 14% to 35% throughout the year, showing no clear difference between the epidemic season and other periods.

Those differences noted in 2023 may reflect, in part, strengthened public health response, including improved case detection, clinical management, and community engagement over the course of 2023 (*13*), along with sensitization efforts before the 2023 epidemic season. Improved clinical awareness and recognition of milder forms of disease, along with faster and more effective management of severe cases, likely contributed to the observed decrease in CFR. However, those interventions were not uniformly implemented across all governorates, and their relative contribution to CFR reduction cannot be quantified in this analysis.

### Factors Associated with Disease Outcome

In Iraq, the logistic regression analysis showed that low Ct value, hemorrhagic symptoms at admission, and age >15 years were independent determinants of a poor CCHF outcome. Our findings are consistent with those of previous studies reporting an association between hemorrhagic symptoms, in particular melena and hematemesis, and fatal outcome (*17*). Systematic standardized clinical data collection and analysis would help assess additional factors that may affect disease outcomes.

### Ct Value as a Key Indicator for Disease Prognosis

Previous studies have indicated association with high viral load (>10^8^copies/mL) and poor outcome (*17*–*19*). Our findings further suggest that low Ct values at admission are also associated with fatal outcome. Similar to the case for Ebola virus disease (*20*) or Lassa fever (*21*), viral load, using Ct value as a proxy, is closely correlated with CCHF outcome. We found a significant difference of 5.6 Ct between median Ct values persons who died from CCHF (median 22.1, range 12.9–37.0) and survivors (median 27.7, range 11.4–38.9), roughly corresponding to a difference in virus RNA concentration of 1.7 log_10_ units (p<0.01). However, further analysis of Ct values across affected countries, considering different CCHF viral genotypes and CFR, is needed.

Among patients with CCHF, those with a Ct value <26 had a 33% CFR, whereas those with a Ct value >26 had a 6% CFR. Between those extremes, Ct values showed a strong negative correlation with CFR. That finding suggests that Ct values are predictive of CCHF CFR and serve as a key indicator of disease prognosis, which is crucial information for clinicians in assessing disease severity and guiding prognosis evaluation. We observed a higher CFR in patients whose Ct values were in the 22–24 range compared with patients whose Ct value was 18–22. A possible explanation is that patients with Ct value of 18–22 had more severe clinical manifestations, prompting more immediate medical intervention, whereas those with Ct values of 22–24 may have exhibited milder initial symptoms, leading to delayed treatment.

Given the strong correlation between Ct values and CCHF CFR, distribution of Ct should be considered when comparing CFRs across districts, governorates, and countries but also across seasons or years. For example, when comparing the CCHF CFR in Iraq between 2022 (20%) and 2023 (14%), standardizing CFR by Ct values can provide more accurate and meaningful comparisons.

Ct values should be interpreted with caution because they are influenced by several factors, including the timing of sample collection relative to symptom onset and underlying viral kinetics. In our analysis, we accounted for those factors by including time from symptom onset to admission in the multivariable model; however, that variable is subject to recall bias and may not fully capture the timing of infection. Therefore, although Ct value at admission appears to be a robust prognostic marker, residual confounding related to disease stage at sampling cannot be excluded.

Ct values can provide a pragmatic and immediately actionable marker for clinicians, particularly in resource-limited settings. They can serve as a useful tool to rapidly assess disease severity at admission and support early clinical decision making, including the timely initiation or intensification of supportive care.

### Lower CCHF CFR in Patients <15 Years of Age 

In our cohort, we observed a 0% mortality rate among the 33 pediatric cases, compared with 17% in adults (p<0.001). Similar trends have been reported in regional studies from Turkey: CFR of 0% (n = 31) (*22*) and 2.1% (n = 47) (*23*) for pediatric cases. Although the biological mechanism for that age-related disparity remains a subject of investigation, our findings reinforce the observation that pediatric CCHF cases often have more favorable clinical outcomes than adult cases. However, those results should be interpreted cautiously and viewed as consistent with regional observations rather than definitive evidence of age-related differences in CFR.

The lower CFR reported in children infected with CCHF in this study contrasts with findings from studies on Ebola virus disease, in which higher CFRs have been documented in younger children, particularly those <5 years of age. That trend was partly attributed to malaria co-infection (*20*) and higher viral loads in pediatric patients (*24*). Conversely, our findings are more consistent with studies on Lassa fever in Nigeria, where children <15 years of age showed lower CFRs than adults (2.9% vs. 13%) (*25*).

Given the rarity of fatal CCHF cases in children in both Iraq and Turkey, a deeper understanding of the clinical and laboratory characteristics of pediatric CCHF is needed. As highlighted previously (*23*), comprehensive multicountry observational studies involving both children and adults are essential. Such studies should investigate host and viral factors that may explain those differences, providing insights into disease progression and pathogenesis.

A limitation of our study is that data were collected retrospectively and collated manually. Although we carefully reviewed the data, we cannot completely rule out the possibility that some mismatches may have occurred. In the different governorates, hospital attendance might have been influenced by factors such as information campaigns, the hospital’s reputation, perceived disease severity, willingness and financial ability to seek treatment and testing, and distance to the healthcare center. Variation in those factors could help explain some of the observed variations in CFR in terms of person, place, and time. Future investigations should also include other circulating genotypes of CCHFV.

In summary, Ct value at admission is a useful indicator of CCHF disease severity to guide clinicians in managing patients; lower Ct values are indicators of poor outcomes. Ct value should be communicated to clinicians to support optimized supportive care for affected patients. 

Appendix Additional information from study of the prognostic value of PCR cycle threshold in Crimean-Congo hemorrhagic fever, Iraq, 2022–2023.
