# Supplementary material for: Prognostic Value of PCR Cycle Threshold Value in Crimean-Congo Hemorrhagic Fever, Iraq, 2022–2023
Source: Emerg Infect Dis. 2026 Jul;32(7):1104–12. doi: 10.3201/eid3207.251284 (PMC13322438; doi:10.3201/eid3207.251284)
Supplement: Appendix — Additional information from study of the prognostic value of PCR cycle threshold in Crimean-Congo hemorrhagic fever, Iraq, 2022–2023. [file 25-1284-Techapp-s1.pdf]

Article DOI: https://doi.org/10.3201/eid3207.251284

EID cannot ensure accessibility for supplementary materials supplied by authors. Readers who have difficulty accessing supplementary content should contact the authors for assistance.

Prognostic Value of PCR Cycle Threshold Value in Crimean-Congo Hemorrhagic Fever, Iraq, 2022–2023

Appendix

Appendix Table. Univariate logistic regression*

Characteristic	N	Event N	OR (95% CI)	p value	q value†
Ct	915	152	0.83 (0.80 to 0.86)	**<0.001**	**<0.001**
Sex	955	156		0.72	0.80
Female			NA		
Male			1.07 (0.75 to 1.52)		
Hemorrhagic	955	156		**<0.001**	**<0.001**
No			NA		
Yes			2.18 (1.54 to 3.09)		
Age	955	156	1.01 (1.00 to 1.02)	**0.041**	0.10
Tick bite	949	155		0.47	0.61
No			NA		
Yes			1.17 (0.76 to 1.78)		
Contact with confirmed case	942	154		0.49	0.61
No			NA		
Yes			0.70 (0.20 to 1.81)		
Contact with raw meat	948	154		0.10	0.20
No			NA		
Yes			1.35 (0.94 to 1.95)		
Contact with animal	950	155		0.87	0.87
No			NA		
Yes			1.03 (0.73 to 1.46)		
Time from onset to admission	950	156	0.95 (0.88 to 1.02)	0.15	0.25
Year of outbreak	955	156		**0.022**	0.072
2022			NA		
2023			0.67 (0.47 to 0.94)		

*Bold text indicates statistical significance after false discovery rate correction. Ct, cycle threshold; NA, not applicable; OR, odds ratio.
†False discovery rate correction for multiple testing.

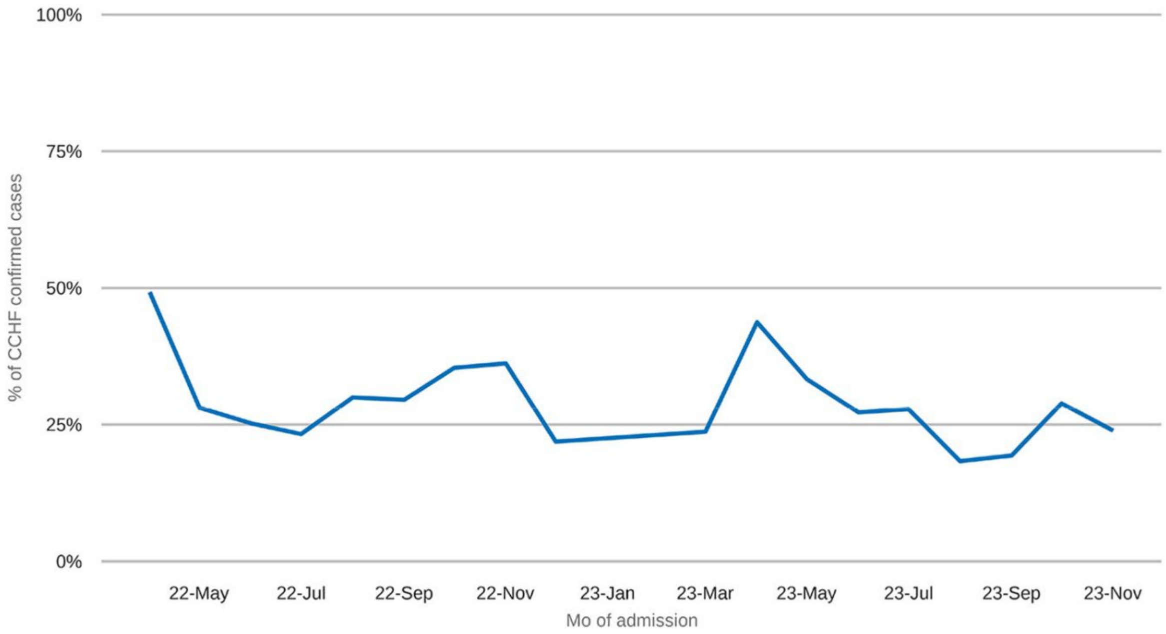

Appendix Figure 1. Rate of CCHF confirmation by month of admission, Iraq, 2022–2023. Rate was calculated using the number of confirmed CCHF patients divided by total number of suspected CCHF patients. For months with less than 5 confirmed cases reported rate of confirmation is not indicated.

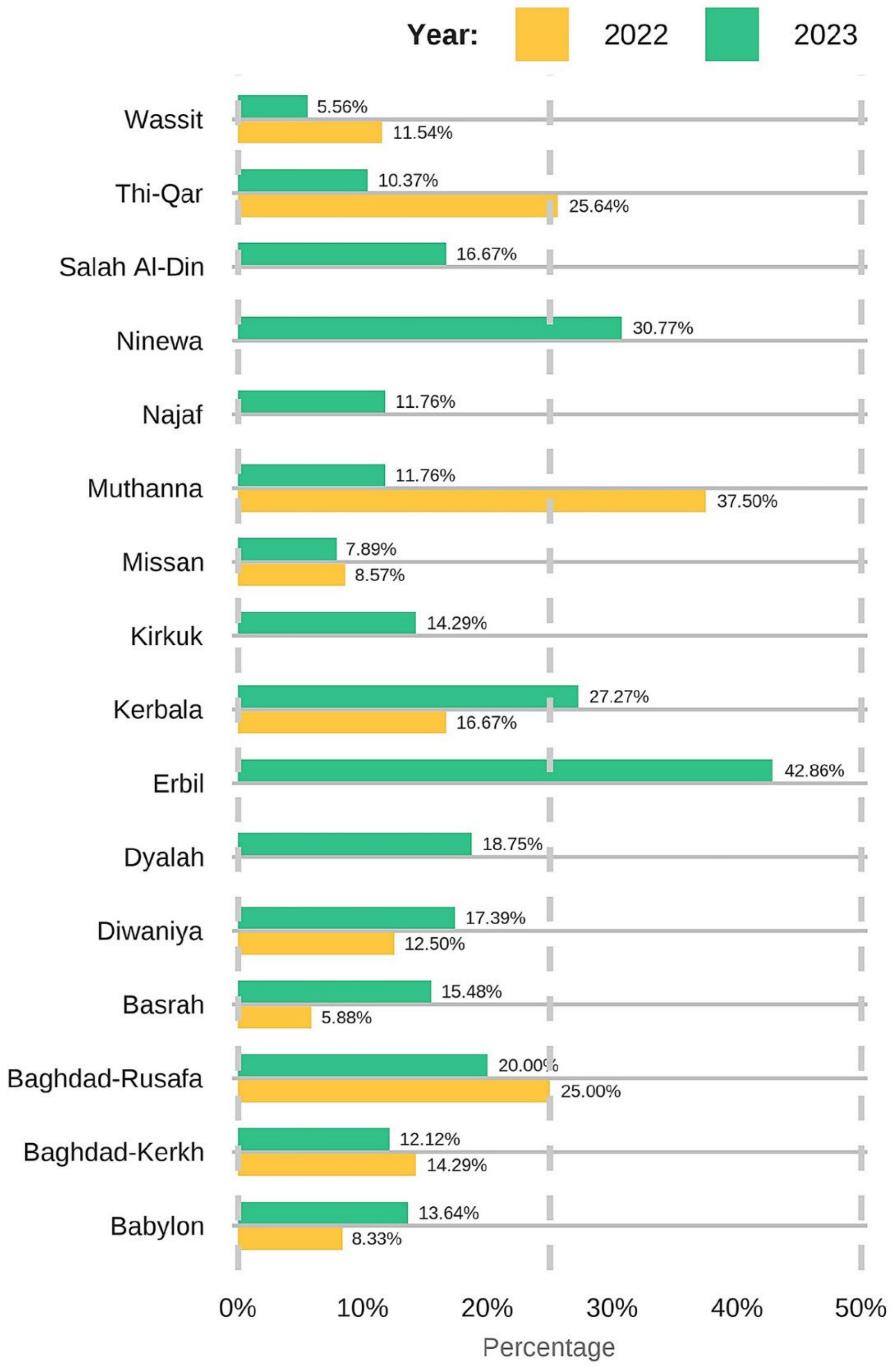

Appendix Figure 2. Case fatality ratio among confirmed CCHF cases by year and Governorate, Iraq, 2022–2023.

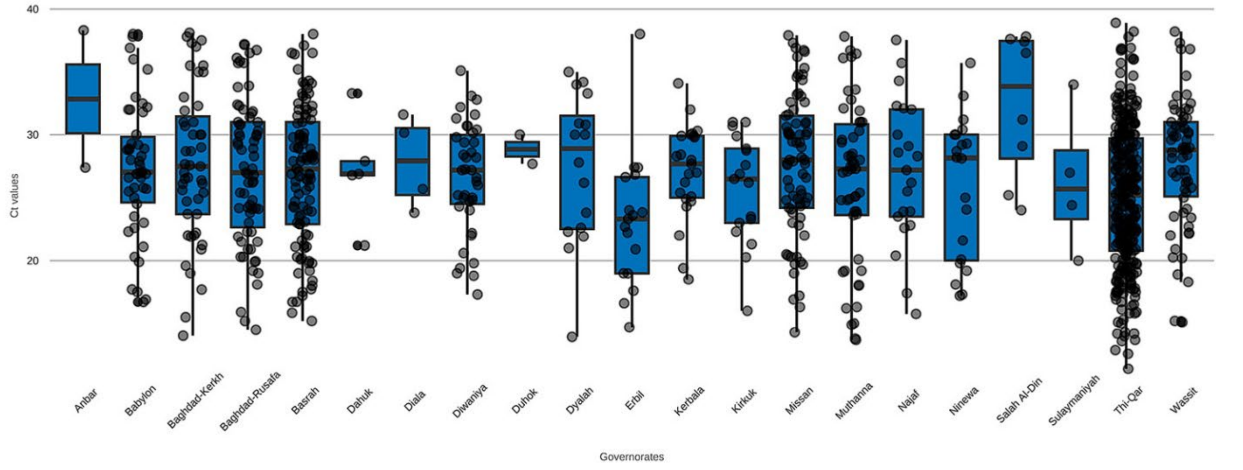

Appendix Figure 3. Distribution of cycle threshold values per governorate, Iraq, 2022–2023.

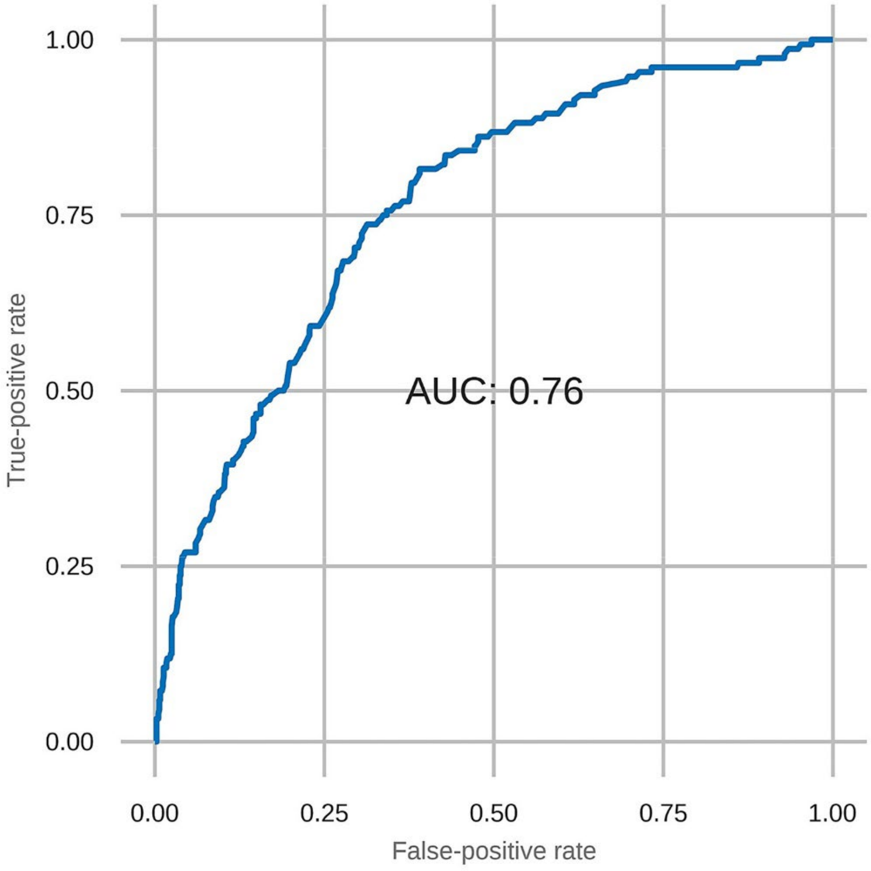

Appendix Figure 4. Receiver operating characteristic curve for patients' outcome and cycle threshold value. With an area under the curve of 0.76, receiver operating characteristic analyses evaluated 26 as the optimal Ct threshold cutoff for predicting patients' outcome (sensitivity 81.6%, specificity 60.9%).

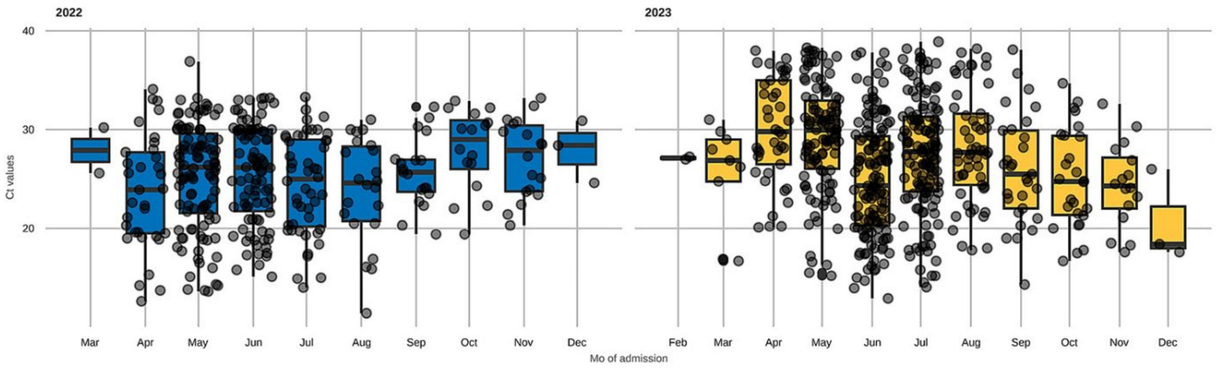

Appendix Figure 5. Distribution of cycle threshold values of CCHF confirmed cases by year and month of admission, Iraq, 2022–2023.

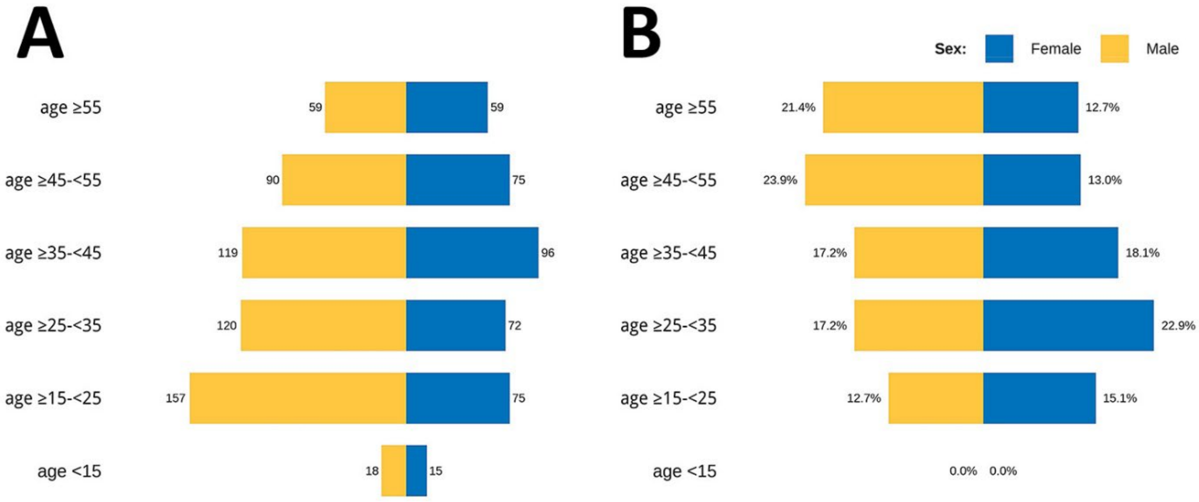

Appendix Figure 6. Confirmed CCHF patients and case fatality ratio by age and sex, Iraq, 2022–2023. A) CCHF confirmed patients distribution by age and sex. B) Case fatality ratio among CCHF confirmed patients by age and sex.

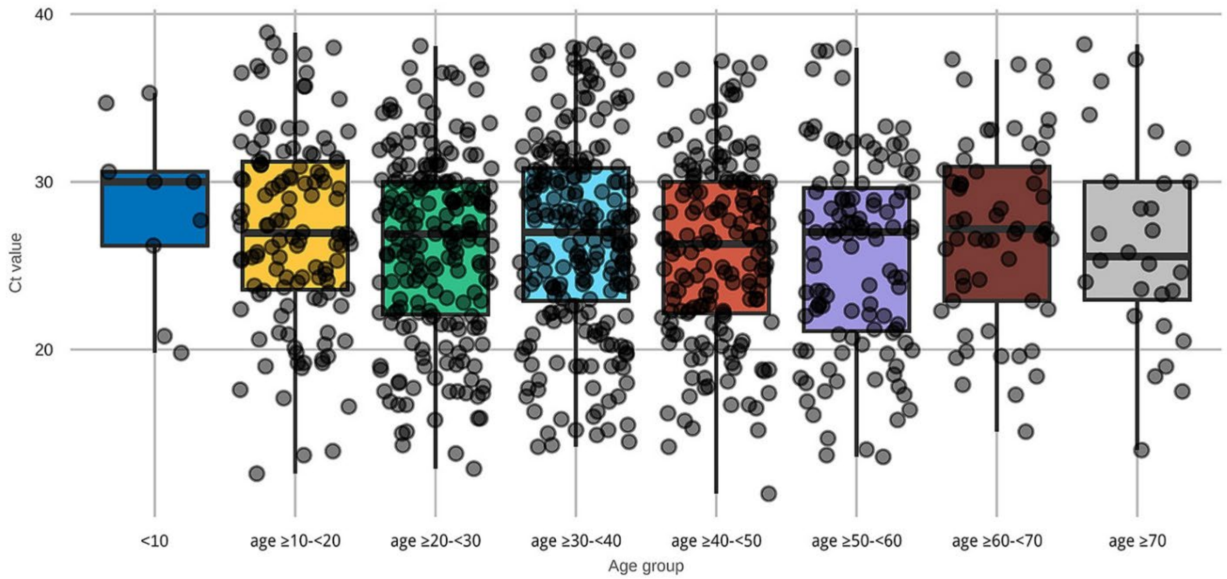

Appendix Figure 7. Distribution of cycle threshold value by age group, Iraq, 2022–2023.
